# A simple technique to reduce evaporation of crystallization droplets by using plate lids with apertures for adding liquids

**DOI:** 10.1107/S2053230X14025126

**Published:** 2014-11-28

**Authors:** Lauren E. Zipper, Xavier Aristide, Dylan P. Bishop, Ishita Joshi, Julia Kharzeev, Krishna B. Patel, Brianna M. Santiago, Karan Joshi, Kahille Dorsinvil, Robert M. Sweet, Alexei S. Soares

**Affiliations:** a Office of Educational Programs, Brookhaven National Laboratory, Upton, NY 11973-5000, USA; b Department of Mechanical Engineering, Binghamton University, 4400 Vestal Parkway East, Vestal, NY 13902, USA; c North Babylon High School, 1 Phelps Lane North, Babylon, NY 11703, USA; d Northport High School, 154 Laurel Hill Road, Northport, NY 11768, USA; e St Augustine Catholic High School, 2188 Rodick Road, Markham, ON L6C 1S3, Canada; f Earl L. Vandermeulen High School, 350 Old Post Road, Port Jefferson, NY 11777, USA; g John P. Stevens High School, 855 Grove Avenue, Edison, NJ 08820, USA; h Connetquot High School, 190 7th Street, Bohemia, NY 11716, USA; i Department of Electronics and Electrical Communication Engineering, PEC University of Technology, Chandigarh, India; j Photon Sciences Directorate, Brookhaven National Laboratory, Upton, NY 11973-5000, USA; k Biosciences Department, Brookhaven National Laboratory, Upton, NY 11973-5000, USA

**Keywords:** crystallization, dehydration, vapor diffusion, high-throughput screening, acoustic droplet ejection, *in situ* X-ray data collection

## Abstract

A method is described for using plate lids to reduce evaporation in low-volume vapor-diffusion crystallization experiments. The plate lids contain apertures through which the protein and precipitants were added to different crystallization microplates (the reservoir was filled before fitting the lids). Plate lids were designed for each of these commonly used crystallization microplates. This system minimizes the dehydration of crystallization droplets containing just a few nanolitres of protein and precipitant, and results in more reproducible diffraction from the crystals. For each lid design, changes in the weight of the plates were used to deduce the rate of evaporation under different conditions of temperature, air movement, droplet size and precipitant. For comparison, the state of dehydration was also visually assessed throughout the experiment. Finally, X-ray diffraction methods were used to compare the diffraction of protein crystals that were conventionally prepared against those that were prepared on plates with plate lids. The measurements revealed that the plate lids reduced the rate of evaporation by 63–82%. Crystals grown in 5 nl drops that were set up with plate lids diffracted to higher resolution than similar crystals from drops that were set up without plate lids. The results demonstrate that plate lids can be instrumental for improving few-nanolitre crystallizations.

## Abstract 2

This article describes the use of evaporation control lids that are fitted to crystallization plates to improve the reproducibility of trials using as little as 5 nl. The plate lids contain apertures which are large enough for the transfer of protein containing droplets, but small enough to greatly reduce the rate of evaporation during the time needed to prepare the plate.

## 1. Introduction   

Dehydration of protein solutions to room air during the time needed to set up vapor-diffusion protein crystallization experiments may compromise the reproducibility of the results, reduce the likelihood of success or degrade the overall quality of the crystals (Zheng *et al.*, 2004 ▶; Rayment, 2002 ▶). When the volume of protein plus precipitant in the crystallization droplet is large compared with the expected evaporative losses, this problem is a nuisance that can be adequately mitigated with simple strategies such as adjusting the ratio of protein to precipitant or preparing the crystallization plates as quickly as possible. However, when the working volume is just a few nanolitres, these palliative measures are no longer adequate (for example, LCP setups under 200 nl require dehydration management; Nollert *et al.*, 2002 ▶). The advent of fourth-generation synchrotron sources with extraordinarily high brightness adds urgency to this problem because the extraordinary brightness of these new X-ray sources may yield high-quality data from very small crystals that can be grown using a few nanolitres of purified protein per tested condition. High-throughput protein crystallization screening efforts such as structural genomics will benefit from nanolitre protein crystallization capabilities (Luft *et al.*, 2011 ▶). The increasing importance of discovery science such as high-throughput screening of fragment libraries will also benefit from small-scale experiments that minimize the consumption of purified protein (Gorrec, 2014 ▶) and accelerate the interaction between crystals and chemicals (Cole *et al.*, 2014 ▶).

One way to prevent desiccation from compromising the quality of micro-crystallization experiments is to choose a strategy that has no liquid–vapor interface (Brumshtein *et al.*, 2008 ▶). A recent review presented three general crystallization strategies, one of which requires a liquid–vapor interface (vapor diffusion) and two which do not (liquid diffusion and batch) (Luft *et al.*, 2014 ▶). When the working volumes are in the vicinity of 1 µl, vapor diffusion accounts for the overwhelming majority of successful crystallization efforts. In contrast, when the working volumes are in the vicinity of 1 nl, batch and liquid-diffusion methods predominate. This contrast is testament to the powerful destructive potential of evaporation to room air during the experimental setup.

Here, we describe a method for using snap-on plate lids with small apertures to greatly reduce dehydration to room air during the preparation of vapor-diffusion-driven protein crystallization experiments. Precipitant is added to the plate reservoir before the plate is covered with a lid. The lid is then fitted and the air spaces above the plate are allowed to equilibrate with the precipitant. The crystallization robot then loads the crystallization shelf through apertures in the plate lid. We designed lids with different sized apertures that are compatible with different crystallization robots. After the setup is complete, the plate lid is replaced with a conventional adhesive sealant. Some crystallization robots have a built-in cover with a similar function (Adachi *et al.*, 2004 ▶; Walter *et al.*, 2003 ▶).

We designed plate lids that fit a variety of crystallization microplates. We then selected four commonly used plate models from different manufacturers and tested the efficacy of our lid designs. For each crystallization plate design, we used a simple strategy to calculate the rate of water loss that affected experiments with and without the plate lids under different environmental conditions and using different common precipitants (we also visually assessed the dehydration of each droplet). We then prepared crystallization microplates using 2.5 nl protein solution and 2.5 nl precipitant solution using an Echo 550 (Labcyte Inc., Sunnyvale, California, USA) acoustic droplet ejection (ADE) instrument. Plates that were covered with a plate lid during preparation were compared with plates that were open to room air while the Echo 550 was loading the plate. In addition to growing crystals using nanolitre volumes (Villaseñor *et al.*, 2012 ▶), ADE can be used for high-throughput screening applications that greatly benefit from nanolitre consumption of purified protein and library chemicals (Yin *et al.*, 2014 ▶; Cuttitta *et al.*, 2015 ▶)^1^. Our results suggest that using plate lids can greatly increase the reproducibility of nanolitre-scale vapor-diffusion experiments.

## 2. Methods   

All of the laboratory work and measurements for this study were performed by high-school and undergraduate interns during the summer of 2014. Interns were mentored collaboratively by personnel from the Photon Sciences Directorate and the Office of Educational Programs^2^.

### 2.1. Designing plate lids and deducing evaporation rates   

**Figure 1:**
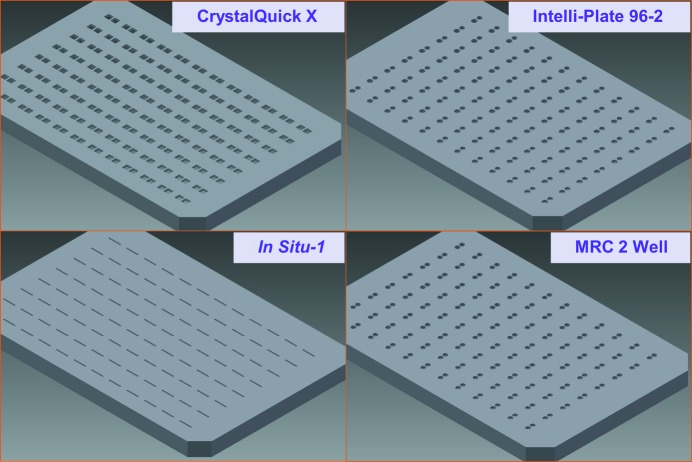
Plate lids. Four plate lids that were designed to fit four popular crystallization plates were tested (similar designs that were not tested are available for many other crystallization plates). The plate lids were constructed out of 1 mm thick acrylonitrile butadiene styrene (Amtek P430) using a three-dimensional printer (Stratasys Dimension Elite).

An undergraduate engineering intern used the *Autodesk Inventor* software to design a series of custom plate lids that snap onto many popular crystallization microplates. Each plate lid completely covers the reservoir and the plate frame, leaving small apertures through which the chemical components were added to the crystallization shelf (Fig. 1 ▶). On average, 93% of the total surface area of the crystallization microplates was sealed by the lids (Table 1 ▶). For each design, three identical plate lids were printed using a three-dimensional printer for testing and for measuring evaporation rates.

**Figure 2:**
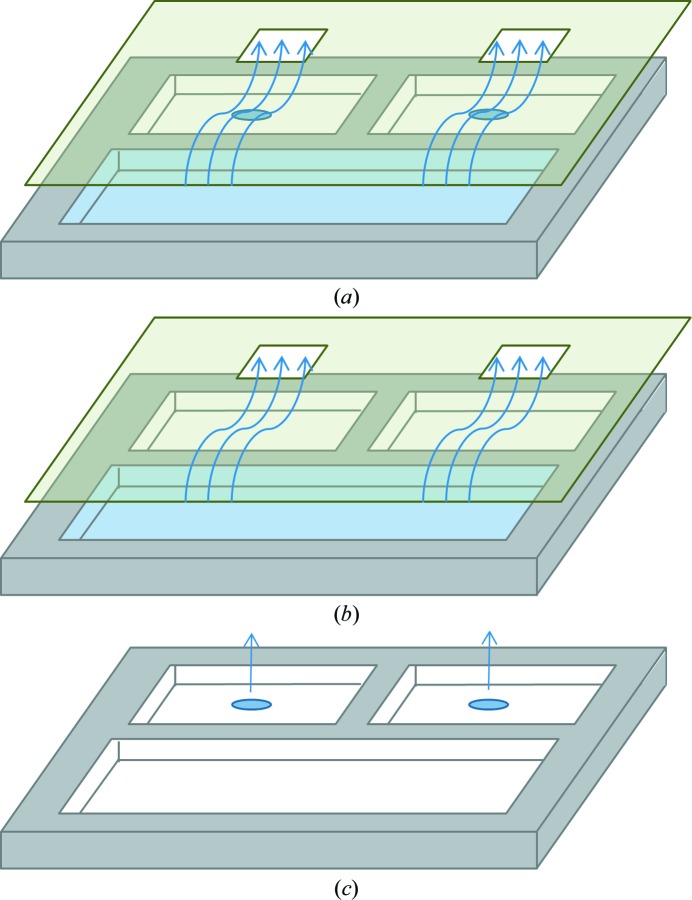
Approach for evaporation control and strategy for calculating the evaporation rate. The plate lids that we designed channel the reservoir vapor pressure over the crystallization shelf in order to shield the crystallization droplet from dehydration by room air (*a*). To deconvolute the evaporation rate of the crystallization droplet from the reservoir, we used an analytical balance to periodically weigh crystallization plates containing either water or three types of mother liquor in two setups as shown in (*a*) and (*b*). We postulated that the small droplet on the crystallization shelf negligibly shielded the larger volume in the reservoir, so that the difference between the two measured evaporation rates was the evaporation rate for the droplet by itself (Evap_shelf_^deduced^ = Evap_A_^Obs^ − Evap_B_^Obs^). We also directly measured the evaporation rate for uncovered plates Evap_C_^Obs^ as shown in (*c*).

The plate lids were designed to channel the equilibrium vapor pressure from the vicinity of the reservoir to the area over the crystallization shelf (Fig. 2 ▶)^3^. This greatly shielded the protein crystallization solution from the dehydration effects of room air. This intentional link between the vapor pressures of the precipitant (in the reservoir) and of the protein crystallization solution (on the shelf) conflates the individual rates of evaporation. To deconvolute the evaporation rates, we periodically measured the total mass of each crystallization plate design in the following three configurations.

(i) Plate + lid with precipitant in the reservoir and protein solution on the shelf (Fig. 2 ▶ *a*).
(ii) Plate + lid with precipitant in the reservoir (Fig. 2 ▶ *b*).
(iii) Plate (no lid) with protein solution on the shelf (Fig. 2 ▶ *c*).

The difference between (i) and (ii) was taken to be the contribution to the overall evaporation rate from the crystallization shelf (using a plate lid)^4^. Measurement (iii) directly yielded the evaporation from the crystallization shelf (with no lid). 

We repeated this measurement strategy for three different starting volumes (2.5, 5.0 and 7.5 µl), for two different environmental conditions (evaporation in room air and under an air stream moving at 0.63 ± 0.10 m s^−1^) and for three different mother-liquor solutions (the mother liquors of lysozyme, trypsin and thaumatin). The evaporation rate was also compared for CrystalQuick plates that were covered with plate lids having apertures of three different sizes. In each case, the size and condition of the solution on the crystallization shelf was simultaneously visually assessed using a Leica MZ16 F microscope (115× maximum magnification). The relative humidity and the temperature in the laboratory were periodically monitored using a sling psychrometer (Sper Scientific, 20–120°F; the air stream mentioned above was taken from room air).

### 2.2. Observing dehydration of nanolitre droplets   

The mass measurements described in §[Sec sec2.1]2.1 verified that plate lids can reduce evaporation rates for solutions of a few microlitres; this does not imply that the lids will be equally effective for solutions of a few nanolitres. However, we could not directly measure the evaporation of few-nanolitre droplets. Instead, we recorded the amount of time required for 2.5 nl water droplets (containing 0.01% methylene blue) to completely evaporate. We manually added 40 µl water to the reservoir of every well in a CrystalQuick X plate. We then used the Echo 550 to dispense 2.5 nl droplets of water (with methylene blue) to crystallization shelves on the plate. We observed each droplet and recorded the elapsed time when each droplet fully evaporated. Using this strategy, we measured the total evaporation time for 20 droplets containing 2.5 nl water and for 20 droplets containing 25 nl water. We then repeated the procedure three times, with the difference that plate lids having apertures of 0.88, 0.66 and 0.44 mm were secured to the plate before dispensing the water droplets with the Echo 550. Additionally, we measured the total evaporation time for an uncovered droplet that was kept at 277 K throughout the experiment (the crystallization plate was pre-cooled to 277 K and the experiment was performed at 277 K).

### 2.3. *In situ* X-ray diffraction   

To complement the evaporation measurements, the diffraction of crystals was assessed *in situ* for crystallization trials conducted with and without plate lids. Lysozyme, trypsin and thaumatin crystals were grown in CrystalQuick X plates (Greiner) using 2.5 nl protein solution plus 2.5 nl precipitant solution; similar experiments were conducted with and without using plate lids (note that the volumes used in §[Sec sec2.1]2.1 were ∼1000 times larger than the volumes used in this section because we could find no instrument with the capability to accurately measure the change in mass of a 5 nl droplet). Conventional adhesive plastic covers were immediately applied to the crystallization plate after the transfer of protein plus precipitant (the plate lids were removed before applying the adhesive). Plates were prepared using an Echo 550 acoustic liquid handler. The Echo 550 uses acoustic droplet ejection (ADE) to eject protein and precipitant solution out of a source plate (5 nl total crystallization droplet), through a short air column and onto the CrystalQuick crystallization microplate. The microplate was incubated at 291 K for 48 h before the number of crystals in each of the 192 crystallization shelves was counted and averaged. We observed the appearance and the number of crystals in each crystallization well (using a Leica microscope).

We obtained X-ray diffraction data from one lysozyme crystal in each of the 12 wells in row *D* of the plate that was prepared with a plate lid and from one lysozyme crystal in each of the 12 wells in row *D* of the plate that was prepared without a plate lid. Data were obtained using a G-rob *in situ* plate-handling system (le Maire *et al.*, 2011 ▶). The diffraction data were obtained by rotating the crystallization plate in the X-ray beam. Row *D* is near the center of the CrystalQuick X plate; optimal data are obtained from this region because the sphere of confusion is minimized and because a larger rotation angle is possible (without the plate colliding with the beamline equipment). For each crystal, 40° of data were obtained (80 rotations of 0.5° with 5 s exposure each). We used *RADDOSE* (Zeldin *et al.*, 2013 ▶) to ensure that the dose was less than 5% of *D*_1/2_ (Owen *et al.*, 2006 ▶). Diffraction data were collected on beamline X12B at the National Synchrotron Light Source (NSLS). Data sets were processed with *HKL*-2000 (Otwinowski & Minor, 2001 ▶) and were further processed using *CTRUNCATE* in the *CCP*4 suite (Winn *et al.*, 2011 ▶). Structures were obtained by molecular substitution (using PDB entry 1lyz; Diamond, 1974 ▶) and refined using *REFMAC* (Winn *et al.*, 2003 ▶) and *ARP*/*wARP* (Perrakis *et al.*, 2001 ▶). Structures were visually inspected using *Coot* (Emsley & Cowtan, 2004 ▶). One benefit of growing crystals in 5 nl is that there was very little room in the droplets for the crystal position to shift during *in situ* data collection.

## 3. Results   

**Figure 3:**
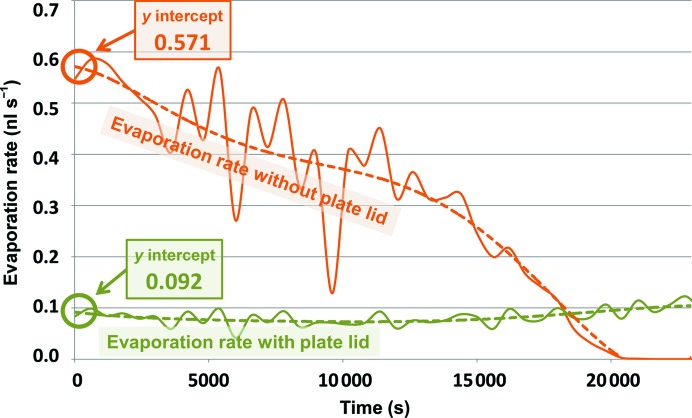
Method for calculating the initial evaporation rate. We periodically measured the evaporation for droplets on covered and uncovered plates. The evaporation rate was calculated for droplets on the crystallization shelf of the uncovered plates and compared with the deduced evaporation rates for droplets on the crystallization shelf of plates covered with lids (using the technique described in §[Sec sec2.1]2.1). These data were plotted as a function of the 6½ h measurement time for each of our tested designs. The initial rate of evaporation was obtained from the *y* intercept of the least-squares fit between a fifth-order polynomial and the evaporation-rate data. The data below were obtained from a 7.5 µl droplet on the crystallization shelf of a CrystalQuick X plate (this data point is boxed in Fig. 4 ▶).

We compared the rate of evaporation from four different plate designs that were covered with plate lids with the rate of evaporation from uncovered plates. For each experiment, the evaporation was measured every 10 min over a period of 6½ h as described in §[Sec sec2.1]2.1. The evaporation rate for plates with no lids was observed to generally decline as a function of time (we postulate that this occurred because the droplets evaporated less as their size decreased). For the plates with lids, this gradual decline was sometimes masked because the droplet size changed much more slowly, and was superposed on a slight increase in evaporation rate (we postulate that this increase occurred because the room temperature rose during the day, causing a slight increase in the evaporation rate). The average air humidity in the experimental area was 65.3 ± 2.4%. The average temperature was 295.7 ± 0.9 K (Supplementary Fig. S1^5^). To illustrate the effect of the plate lids in a uniform way, we fitted each evaporation-rate data set with a fifth-order polynomial using least squares. Consequently, the *y* intercept signified the initial evaporation rate at time zero (Fig. 3 ▶).

### 3.1. Mass measurements   

**Figure 4:**
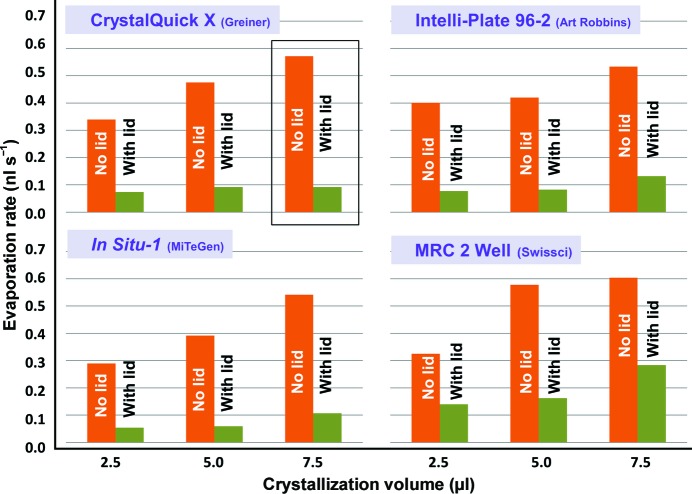
Plate lids reduce the rate of evaporation. Rate values are the initial rates of evaporation determined from the *y* intercept of a fifth-order polynomial that was least-squares fitted to each data set as described in Fig. 3 ▶ (the data used in Fig. 3 ▶ to illustrate the method for determining the *y* intercept are boxed).

Fig. 4 ▶ shows that using plate lids effectively reduced the rate of evaporation for all of the designs that we tested. On average, the plate lids reduced the rate of evaporation of 2.5–7.5 µl droplets of water that were positioned on the crystallization shelf by 77% (81% for Greiner plates, 83% for MiTeGen plates, 79% for Intelli-Plates and 63% for MRC plates).

**Figure 5:**
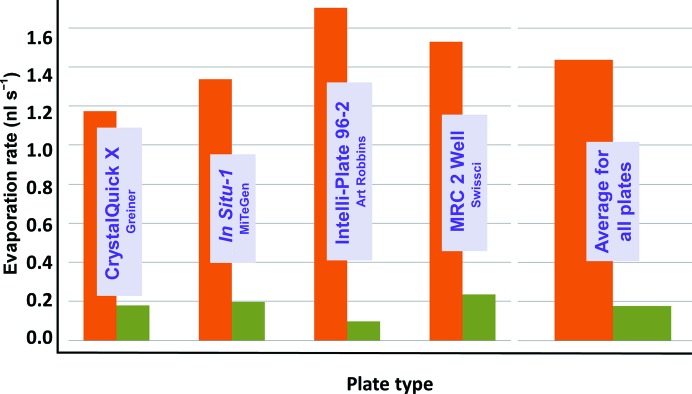
The effect of air currents on evaporation. Air currents (taken from room air) greatly increase the evaporation rate for uncovered plates. The evaporation rates for all tested plates that were not fitted with lids (shown in orange) were much larger than the evaporation rates for the same plates with lids (shown in green).

Fig. 5 ▶ illustrates the impact on the evaporation rate caused by a 0.63 ± 0.10 m s^−1^ laminar air flow over each type of plate. Moving air increased the average evaporation rate of 2.5 µl droplets in the four uncovered tested plate types by 220% (from 0.45 to 1.44 nl s^−1^ ). When an air current was directed over a plate with a lid, the evaporation rate also increased, but not as much (by 57%, from 0.11 to 0.18 nl s^−1^).

**Figure 6:**
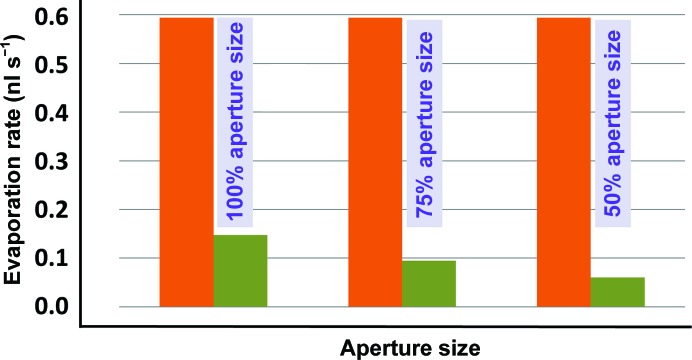
Smaller apertures reduce evaporation rate. We compared the measured evaporation rate for uncovered plates (orange) with the evaporation rate for plates that were covered with plate lids that had square apertures with 0.88 mm sides (left), 0.66 mm sides (middle) and 0.44 mm sides (right). The smaller apertures further reduced the deduced rates of evaporation (green) at the cost of requiring greater precision from the plate-preparation robot.

Fig. 6 ▶ compares the observed and deduced evaporation rates for CrystalQuick plates that were covered with plate lids that had square apertures with side lengths of 0.88, 0.66 and 0.44 mm (100, 75 and 50% of our original design size). The results show that smaller apertures further reduce the deduced rates of evaporation. To use the smallest sized apertures, the Echo 550 had to be carefully programmed to prevent the ejected droplet from inadvertently missing the aperture (larger holes were much more forgiving of instrument error).

### 3.2. Evaporation time trials   

For 2.5 nl water droplets deposited on a CrystalQuick X plate, the average time needed for total evaporation was 40 s. The total evaporation time was increased by 150% when the plate was covered with a plate lid with 0.88 mm apertures. The total evaporation time was further doubled when the size of the apertures was decreased to 0.44 mm (see Table 2 ▶). For 25 nl water droplets, the average time needed for total evaporation was 216 s and the plate lids were similarly effective. When the experiment was repeated at 277 K the total evaporation time was tripled. Using a plate lid while also at 277 K amplified both effects. This underscores the effectiveness of working in a cold room to control evaporation (for crystals that tolerate low temperatures).

### 3.3. Crystal characterization and *in situ* X-ray diffraction   

**Figure 7:**
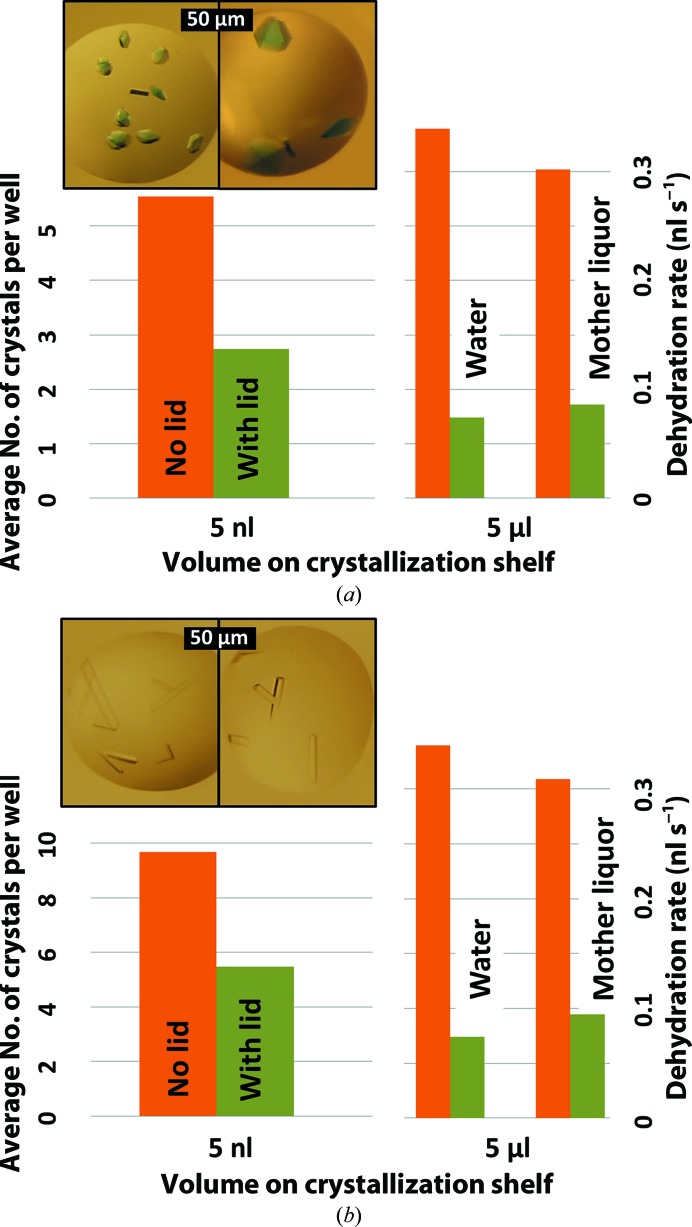
Plate lids result in better and more reproducible crystals. The figure shows crystals that were grown using 2.5 nl precipitant solution and 2.5 nl lysozyme (*a*) and trypsin (*b*) solution in CrystalQuick plates (approximately 2½ min for the Echo 550 to prepare each plate). Each type of protein was prepared on a plate that had a lid and on a plate that did not have a plate lid. On average, using a plate lid resulted in fewer but larger crystals of lysozyme and trypsin (left). The number of thaumatin crystals was approximately constant. X-ray data were obtained from one of the lysozyme crystals in each well in the middle row of the plate (row *D*). The data from crystals prepared using lids were merged and compared with the data from crystals prepared without lids. We also used the technique described in §[Sec sec2.1]2.1 to measure the evaporation rate for each of the three types of mother liquor with no plate lid, and compared this with the deduced evaporation rate with a plate lid (right). Using lids during the 2½ min required for the Echo 550 to set up the 5 nl crystallization droplets allowed better, more reproducible and better diffracting crystals to be obtained.

To further demonstrate the efficiency of the plate lids, we compared the size and diffraction of crystals that were prepared with and without plate lids. We observed that lysozyme and trypsin crystals that were prepared in trays without a lid were smaller and more numerous than the crystals that were prepared in trays with a lid (Fig. 7 ▶ inset, left). On average, there were twice as many lysozyme and trypsin crystals in the 192 wells of plates that were prepared without plate lids compared with plates there were prepared with plate lids (5.54 *versus* 2.74 lysozyme crystals, 9.68 *versus* 5.48 trypsin crystals; Fig. 7 ▶). There was no difference in the average number of thaumatin crystals observed. We also measured the evaporation rate of 2.5 µl mother liquor using the technique that was described in §[Sec sec2.1]2.1 (Fig. 7 ▶, right).

We merged X-ray diffraction data from 12 lysozyme crystals that were prepared on a plate with a lid, and compared these data with similar data merged from 12 lysozyme crystals that were prepared on a plate without a lid (data were obtained from one crystal in each of the 12 wells in row *D*). The average resolution at *I*/σ(*I*) = 1.0 was 1.55 ± 0.09 Å for lysozyme crystals that were prepared without a lid, compared with 1.44 ± 0.08 Å for lysozyme crystals that were prepared with a lid (see Table 3 ▶). These results demonstrate that crystallization conditions that are optimal for larger crystallization volumes can be extended to 5 nl drops when lids are used to reduce evaporation.

It is likely that the improved diffraction that we observed from lysozyme crystals that were prepared using plate lids was largely because these crystals were fewer in number and larger in size compared with similar crystals that were prepared without plate lids. Larger crystals have a better signal to noise at high angle, which is probably why the resolution was improved. Hence, using plate lids allows the protein and precipitant concentrations in few-nanolitre crystallization drops to more closely resemble the concentrations in few-microlitre crystallization drops (small volumes may introduce other discrepancies such as the number and frequency of nucleation events).

## 4. Discussion   

Protein crystallization on the nanolitre scale has obvious advantages for structure-determination efforts that have limited quantities of available purified protein (Gorrec, 2014 ▶). Nanolitre crystallization can also facilitate high-throughput screening applications such as fragment-based drug discovery, making such research accessible to academic laboratories with limited resources. These advantages have motivated numerous efforts to probe crystallization in a few nanolitres with various different technologies. However, desiccation of the protein solution by room air during the preparation of the crystallization microplate setup has complicated micro-crystallization with conventional vapor diffusion, and most of the successful nanolitre crystallization technologies exploit evaporation-free strategies such as microfluidic diffusive mixing techniques (Trastoy *et al.*, 2013 ▶; Zheng *et al.*, 2004 ▶) or microbatch applications under oil (Zhu *et al.*, 2014 ▶; Kisselman *et al.*, 2011 ▶). The great success of vapor-diffusion crystallization strategies in the microlitre regime suggests that the fact that nanolitre crystallization strategies cluster in technologies where there is no liquid–vapor interface is an artifact of the destructive effect of dehydration to room air. With proteins that are stable at 277 K, one option is to position the crystallization robot in a cold room. If cold operation is not possible, evaporation can be significantly reduced by preparing the crystallization experiment using apertures. Using plate lids, we have demonstrated that conventional vapor-diffusion crystallization is viable at room temperature using 2.5 nl purified protein solution and 2.5 nl precipitant solution.

## Supplementary Material

Supporting Information.. DOI: 10.1107/S2053230X14025126/nj5207sup1.pdf

## Figures and Tables

**Table 1:** Testing results for plate-lid designs The measurements shown in the top four rows were performed in an educational outreach building with limited climate control (average humidity 65%, average temperature 295.7K; see Supplementary Fig. S1). The measurements shown in the bottom three rows were performed in a laboratory with constant humidity (50%) and constant temperature (297K).

	Aperture size (per well)	Percentage of total surface area covered by lid	Average reduction in evaporation rate (%)
CrystalQuick X	0.88 0.88mm	91	81
*In situ*-1	1.1 0.24mm	94	82
Intelli-Plate 96-2	*r* = 0.45mm	93	79
MRC 2 Well	*r* = 0.375mm	95	63
CrystalQuick X (100%)	0.88 0.88mm	91	75
CrystalQuick X (75%)	0.66 0.66mm	95	84
CrystalQuick X (50%)	0.44 0.44mm	98	90

**Table 2:** Time needed for total evaporation The average time required for total evaporation of a 2.5 or a 25nl droplet of water (with 0.01% methylene blue) under different circumstances is shown.

Droplet size (nl)	2.5	25	25
Temperature (K)	297	297	277
No lid (s)	40 7	216 28	611 51
CrystalQuick X (0.88mm) (s)	104 54	596 90	1547 115
CrystalQuick X (0.66mm) (s)	159 61	891 96	
CrystalQuick X (0.44mm) (s)	198 83	1083 128	

**Table 3:** Data-collection and refinement parameters Diffraction data were obtained *in situ* from lysozyme crystals that were grown using 2.5nl protein solution and 2.5nl precipitant solution. Data were obtained from 12 crystals that were grown on a plate that was prepared with a plate lid and from 12 crystals that were grown on a plate that was prepared without a plate lid. The top of the table shows the data-collection statistics for each group of 12 data sets (average standard deviation). The bottom of the table shows the merging statistics and refinement statistics after merging each group of 12 data sets.

	With lid	Without lid
Crystal information		
No. of crystals	12	12
Space group	*P*4_3_2_1_2	*P*4_3_2_1_2
Unit-cell parameters ()		
*a* = *b*	79.573 0.118	79.563 0.085
*c*	37.854 0.075	37.843 0.099
Data-collection statistics (average of 12 unmerged data sets)		
Resolution ()	1.44 0.08	1.55 0.09
Unique reflections	18255 3186	15060 2525
*R*_sym_ (%)	5.8 0.9	7.6 1.4
Merging statistics (merged data)		
Resolution ()	1.4	1.5
Unique reflections	24370	19689
*R*_merge_ (%)	9.6	11.6
Refinement statistics (merged data)		
*R*_work_ (%)	15.1	14.6
*R*_free_ (%)	16.8	17.1
R.m.s.d., bond lengths ()	0.029	0.027
R.m.s.d., bond angles ()	2.53	2.33

## Footnotes

1 ADE can also be used to harvest very small crystals (Soares *et al.*, 2011 ▶; Roessler *et al.*, 2013 ▶).

2 We leveraged this project to develop a model for integrating science with education in a way that maximizes both the research value to the institution and the educational value to the community. One key ingredient was the close collaboration between the scientist who originates the project and the program directors in the Office of Education Programs. This study was selected as a pilot project because the experimental techniques were uncomplicated, because the number of measurements that had to be made was very large and because the project required minimal equipment and infrastructure. We hope that the future availability of a dedicated interface between Brookhaven National Laboratory and the Long Island community (called the Portal to Discovery) will provide growing opportunities for citizen science initiatives and also will amplify the scope of projects that are technically accessible.

3 The reservoir contained either water (with 0.01% methylene blue for easy visualization) or one of three types of mother liquor.

4 We cannot directly measure the change in the mass of the droplet on the crystallization shelf independently of the change in mass of the reservoir. Instead, we assume that the small volume on the crystallization shelf provides a negligible reduction in the evaporation rate for the much larger reservoir. Consequently, by separately measuring the change in mass of two plates (one having a filled crystallization area and one having an empty crystallization area), we deduced the evaporation rate of the droplet on the crystallization shelf by subtracting the reservoir evaporation rate (Fig. 2 ▶ *b*) from the reservoir + shelf evaporation rate (Fig. 2 ▶ *a*). We visually monitored the appearance of the droplet on the crystallization shelf to corroborate this calculation.

5 Supporting information has been deposited in the IUCr electronic archive (Reference: NJ5207).

## References

[bb1] Adachi, H., Takano, K., Matsumura, H., Niino, A., Ishizu, T., Inoue, T., Mori, Y. & Sasaki, T. (2004). *Jpn. J. Appl. Phys.* **43**, l76–L78.

[bb2] Brumshtein, B., Greenblatt, H. M., Futerman, A. H., Silman, I. & Sussman, J. L. (2008). *J. Appl. Cryst.* **41**, 969–971. 10.1107/S0021889808024667 PMC2553557 19461852

[bb3] Cole, K. *et al.* (2014). *PLoS One*, **9**, e101036. 10.1371/journal.pone.0101036 PMC4079544 24988328

[bb4] Cuttitta, C. M., Ericson, D. L., Scalia, A., Roessler, C. G., Teplitsky, E., Joshi, K., Campos, O., Agarwal, R., Allaire, M., Orville, A. M., Sweet, R. M. & Soares, A. S. (2015). *Acta Cryst.* D**71**, XXX–XXX. 10.1107/S1399004714013728 PMC4304690 25615864

[bb5] Diamond, R. (1974). *J. Mol. Biol.* **82**, 371–391. 10.1016/0022-2836(74)90598-1 4856347

[bb6] Emsley, P. & Cowtan, K. (2004). *Acta Cryst.* D**60**, 2126–2132. 10.1107/S0907444904019158 15572765

[bb7] Gorrec, F. (2014). *Drug Discov. Today*, **19**, 1505–1507 10.1016/j.drudis.2014.07.002 PMC4874463 25048082

[bb8] Kisselman, G., Qiu, W., Romanov, V., Thompson, C. M., Lam, R., Battaile, K. P., Pai, E. F. & Chirgadze, N. Y. (2011). *Acta Cryst.* D**67**, 533–539. 10.1107/S0907444911011589 PMC3107051 21636893

[bb9] Luft, J. R., Newman, J. & Snell, E. H. (2014). *Acta Cryst.* F**70**, 835–853. 10.1107/S2053230X1401262X PMC4089519 25005076

[bb10] Luft, J. R., Snell, E. H. & DeTitta, G. T. (2011). *Expert Opin. Drug. Discov.* **6**, 465–480. 10.1517/17460441.2011.566857 PMC3637395 22646073

[bb11] Maire, A. le, Gelin, M., Pochet, S., Hoh, F., Pirocchi, M., Guichou, J.-F., Ferrer, J.-L. & Labesse, G. (2011). *Acta Cryst.* D**67**, 747–755. 10.1107/S0907444911023249 21904027

[bb12] Nollert, P., Navarro, J. & Landau, E. M. (2002). *Methods Enzymol.* **343**, 183–199. 10.1016/s0076-6879(02)43135-7 11665567

[bb13] Otwinowski, Z. & Minor, W. (2001). *International Tables for Crystallography*, Vol. *F*, edited by M. G. Rossmann & E. Arnold, pp. 226–235. Dordrecht: Kluwer Academic Publishers.

[bb14] Owen, R. L., Rudiño-Piñera, E. & Garman, E. F. (2006). *Proc. Natl Acad. Sci. USA*, **103**, 4912–4917. 10.1073/pnas.0600973103 PMC1458769 16549763

[bb15] Perrakis, A., Harkiolaki, M., Wilson, K. S. & Lamzin, V. S. (2001). *Acta Cryst.* D**57**, 1445–1450. 10.1107/s0907444901014007 11567158

[bb16] Rayment, I. (2002). *Structure*, **10**, 147–151. 10.1016/s0969-2126(02)00711-6 11839300

[bb17] Roessler, C. G., Kuczewski, A., Stearns, R., Ellson, R., Olechno, J., Orville, A. M., Allaire, M., Soares, A. S. & Héroux, A. (2013). *J. Synchrotron Rad.* **20**, 805–808. 10.1107/S0909049513020372 PMC3747951 23955046

[bb18] Soares, A. S., Engel, M. A., Stearns, R., Datwani, S., Olechno, J., Ellson, R., Skinner, J. M., Allaire, M. & Orville, A. M. (2011). *Biochemistry*, **50**, 4399–4401. 10.1021/bi200549x PMC3144476 21542590

[bb19] Trastoy, B., Lomino, J. V., Wang, L.-X. & Sundberg, E. J. (2013). *Acta Cryst.* F**69**, 1405–1410. 10.1107/S1744309113030650 PMC3855731 24316841

[bb20] Villaseñor, A. G., Wong, A., Shao, A., Garg, A., Donohue, T. J., Kuglstatter, A. & Harris, S. F. (2012). *Acta Cryst.* D**68**, 893–900. 10.1107/S0907444912016617 PMC3413209 22868754

[bb21] Walter, T. S., Diprose, J., Brown, J., Pickford, M., Owens, R. J., Stuart, D. I. & Harlos, K. (2003). *J. Appl. Cryst.* **36**, 308–314.

[bb22] Winn, M. D. *et al.* (2011). *Acta Cryst.* D**67**, 235–242.

[bb23] Winn, M. D., Murshudov, G. N. & Papiz, M. Z. (2003). *Methods Enzymol.* **374**, 300–321. 10.1016/S0076-6879(03)74014-2 14696379

[bb24] Yin, X., Scalia, A., Leroy, L., Cuttitta, C. M., Polizzo, G. M., Ericson, D. L., Roessler, C. G., Campos, O., Ma, M. Y., Agarwal, R., Jackimowicz, R., Allaire, M., Orville, A. M., Sweet, R. M. & Soares, A. S. (2014). *Acta Cryst.* D**70**, 1177–1189. 10.1107/S1399004713034603 PMC4014116 24816088

[bb25] Zeldin, O. B., Gerstel, M. & Garman, E. F. (2013). *J. Appl. Cryst.* **46**, 1225–1230.

[bb26] Zheng, B., Tice, J. D., Roach, L. S. & Ismagilov, R. F. (2004). *Angew. Chem. Int. Ed.* **43**, 2508–2511. 10.1002/anie.200453974 PMC1766324 15127437

[bb27] Zhu, Y., Zhu, L. N., Guo, R., Cui, H. J., Ye, S. & Fang, Q. (2014). *Sci. Rep.* **4**, 5046. 10.1038/srep05046 PMC5154416 24854085

