# Supplementary material for: A simple technique to reduce evaporation of crystallization droplets by using plate lids with apertures for adding liquids
Source: Acta Crystallogr F Struct Biol Commun. 2014 Nov 28;70(Pt 12):1707–13. doi: 10.1107/S2053230X14025126 (PMC4259245; doi:10.1107/S2053230X14025126)
Supplement: Supplementary file 1 [file f-70-01707-sup1.pdf]

STRUCTURAL BIOLOGY COMMUNICATIONS

Volume 70 (2014)

Supporting information for article:

A simple technique to reduce evaporation of crystallization droplets by using plate lids with apertures for adding liquids

Lauren E. Zipper, Xavier Aristide, Dylan P. Bishop, Ishita Joshi, Julia Kharzeev, Krishna B. Patel, Brianna M. Santiago, Karan Joshi, Kahille Dorsinvil, Robert M. Sweet and Alexei S. Soares

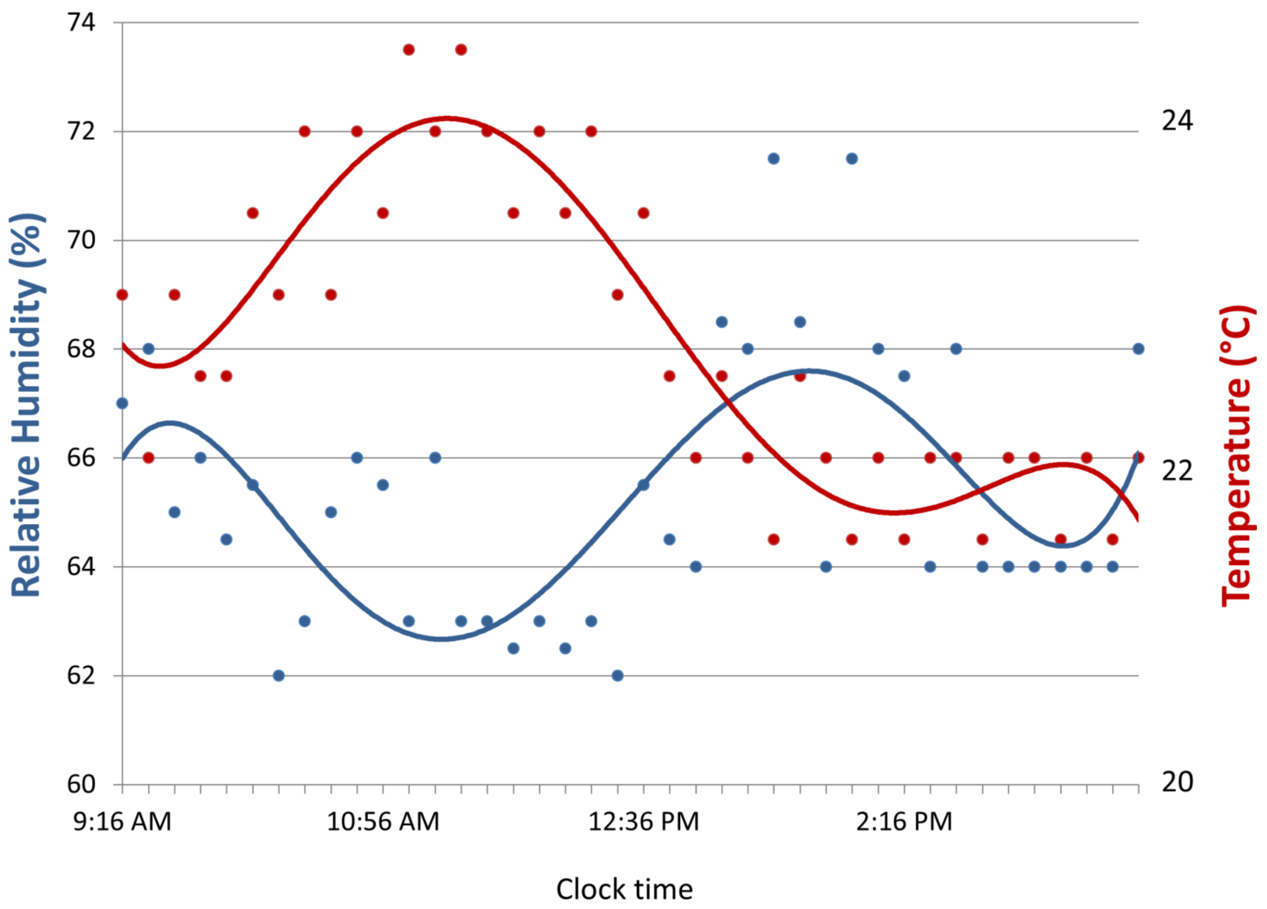

Figure S1 Graph of relative humidity (blue) and temperature (red) in the education outreach facility during one of our measurement days. Some of the measurements were also made in a laboratory with strictly controlled climate.
